# Supplementary material for: Surveying the Side-Chain Network Approach to Protein Structure and Dynamics: The SARS-CoV-2 Spike Protein as an Illustrative Case
Source: Front Mol Biosci. 2020 Dec 18;7:596945. doi: 10.3389/fmolb.2020.596945 (PMC7775578; doi:10.3389/fmolb.2020.596945)
Supplement: Supplementary file 1 [file Table_1.DOCX]

**Table S1**: Sorted clusters obtained from Feidler Vector Components (FVC) for the RBD domain of the SARS-Cov2 spike protein (PDB_id: 6LZG).

| **Cluster sorted_Size** | **Node** | **FVC** | **Interface** |
| --- | --- | --- | --- |
| **1** | ALA397 | -0.030522094 |  |
|  | VAL341 | -0.030522094 |  |
|  | SER399 | -0.030522094 |  |
|  | PHE347 | -0.030522094 |  |
|  | TYR453 | -0.030522094 | Interface |
|  | TYR495 | -0.030522094 |  |
|  | ARG509 | -0.030522094 |  |
|  | PHE497 | -0.030522094 |  |
|  | SER438 | -0.030522094 |  |
|  | PRO507 | -0.030522094 |  |
|  | ASN439 | -0.030522094 |  |
|  | SER443 | -0.030522094 |  |
|  | PRO499 | -0.030522094 |  |
|  | ASP442 | -0.030522094 |  |
|  | GLN506 | -0.030522094 |  |
|  | THR376 | -0.030522094 |  |
|  | ASN437 | -0.030522094 |  |
|  | GLY404 | -0.030522094 |  |
|  | VAL407 | -0.030522094 |  |
|  | TYR508 | -0.030522094 |  |
|  | ALA435 | -0.030522094 |  |
|  | TYR451 | -0.030522094 |  |
|  | VAL401 | -0.030522094 |  |
|  | VAL510 | -0.030522094 |  |
|  | PHE400 | -0.030522094 |  |
|  | VAL350 | -0.030522094 |  |
|  | ASN422 | -0.030522094 |  |
|  | ILE402 | -0.030522094 |  |
|  | GLU406 | -0.030522094 |  |
|  | ARG403 | -0.030522094 |  |
|  | ILE418 | -0.030522094 |  |
|  | GLN409 | -0.030522094 |  |
|  | ALA419 | -0.030522094 |  |
|  | GLY416 | -0.030522094 |  |
|  | ASP420 | -0.030522094 |  |
|  | ASN460 | -0.030522094 |  |
| **2** | ILE434 | 0.018139616 |  |
|  | PHE377 | 0.018139616 |  |
|  | TYR369 | 0.018139616 |  |
|  | PHE374 | 0.018139616 |  |
|  | CYS432 | 0.018139616 |  |
|  | CYS379 | 0.018139616 |  |
|  | PRO384 | 0.018139616 |  |
|  | VAL382 | 0.018139616 |  |
|  | SER383 | 0.018139616 |  |
|  | LEU387 | 0.018139616 |  |
| 3A | TYR396 | 0.044163446 |  |
|  | ARG355 | 0.044163446 |  |
|  | ASP398 | 0.044163446 |  |
|  | TRP353 | 0.044163446 |  |
|  | ARG466 | 0.044163446 |  |
|  | TYR423 | 0.044163446 |  |
| 3B | PRO521 | -0.002079435 |  |
|  | CYS525 | -0.002079435 |  |
|  | CYS391 | -0.002079435 |  |
|  | THR393 | -0.002079435 |  |
|  | ALA522 | -0.002079435 |  |
|  | ALA520 | -0.002079435 |  |
| 3C | ASN501 | -0.108132187 |  |
|  | GLN498 | -0.108132187 | Interface |
|  | GLY447 | -0.108132187 |  |
|  | TYR449 | -0.108132187 | Interface |
|  | SER494 | -0.108132187 |  |
|  | GLY496 | -0.108132187 | Interface |
| 4 | TYR365 | -0.045666956 |  |
|  | ALA363 | -0.045666956 |  |
|  | VAL524 | -0.045666956 |  |
|  | CYS361 | -0.045666956 |  |
|  | CYS336 | -0.045666956 |  |
| 5A | PRO463 | 0.106676295 |  |
|  | PRO426 | 0.106676295 |  |
|  | PHE464 | 0.106676295 |  |
|  | SER514 | 0.106676295 |  |
| 5B | ARG454 | 0.24431682 |  |
|  | SER469 | 0.24431682 |  |
|  | ASP467 | 0.24431682 |  |
|  | ARG457 | 0.24431682 |  |
| 5C | GLY485 | -0.213268609 |  |
|  | CYS480 | -0.213268609 |  |
|  | CYS488 | -0.213268609 |  |
|  | ILE472 | -0.213268609 |  |
| 6 | VAL512 | -0.19213586 |  |
|  | ILE410 | -0.19213586 |  |
|  | VAL433 | -0.19213586 |  |
|  | GLU465 | -0.176501094 |  |
|  | LEU461 | -0.176501094 |  |
|  | LYS462 | -0.176501094 |  |
|  | GLY476 | 0.079998169 |  |
|  | ASN487 | 0.079998169 | Interface |
|  | ALA475 | 0.079998169 |  |
|  | LEU425 | 0.245773266 |  |
|  | PHE429 | 0.245773266 |  |
|  | GLY431 | 0.245773266 |  |
|  | PHE490 | 0.002919916 |  |
|  | TYR351 | 0.002919916 |  |
|  | LEU492 | 0.002919916 |  |
|  | VAL362 | 0 |  |
|  | GLY526 | 0 |  |
|  | PRO527 | 0 |  |

**Table S2:** Details of the dynamically stable common hub residues in both the closed (PDB_ID: 6VXX) and partially open (PDB_ID: 6YVB) conformational states of the trimeric SARS-CoV-2 spike protein. Structural depiction of the dynamically stable hub residues unique to the closed and partially open conformational states of the SARS-CoV-2 spike protein is shown in Figure 5.

| Structural Domains of SARS-cov2 | **Subunits of SARS-cov2** | | |
| --- | --- | --- | --- |
|  | **Chain A** | **Chain B** | **Chain C** |
| **NTD (32-324)** | 34, 37, 43, 86, 91, 92, 104, 106, 194, 195, 201, 238, 265, 290, 306, 318 | 34, 37, 43, 44, 81, 86, 104, 106, 191, 194, 195, 201, 240, 265, 266, 306, 315, 319 | 34, 37, 43, 59, 81, 86, 91, 92, 104, 106, 194, 195, 201, 238, 240, 265, 290 |
| **NTD-RBD linker (325-337)** | 328 | 328 | 328 |
| **RBD (338-560)** | 342, 353, 400, 406, 423, 429, 436, 454, 457 464, 507, 509, 515 | 342, 353, 358, 400, 423, 429, 442, 451, 454, 457, 464, 465, 507, 509, 515, 544, 559 | 342, 353, 395, 396, 400, 403, 423, 429, 442, 451, 454, 457, 507, 509, 515, 544 |
| **SD1 (561-632)** | 592, 612, 674 | 597, 612 | 612 |

| **SD2 (633-704)** | 674 | 0 | 651 |
| --- | --- | --- | --- |
| **S2 (705-930)** | 718, 740, 741, 749, 756, 759, 802, 819, 898, 900, 901, 904, 906, 917, 927 | 718, 740, 741, 756, 797, 802, 819, 898, 900, 906, 913, 914, 917, 927 | 718, 731, 741, 759, 797, 802, 819, 898, 901, 906, 917 |
| **HR1 (931-1003)** | 965 | 965 | 965 |
| **HR1-HR2 linker (1004-1181)** | 1005, 1007, 1028, 1029, 1039, 1050, 1054, 1064, 1067, 1089, 1095 | 1005, 1007, 1028, 1029, 1039, 1050, 1054, 1064, 1067, 1089, 1095 | 1005, 1007, 1028, 1029, 1039, 1047, 1054, 1064, 1067, 1089, 1095, 1102, 1132 |

**Table S3a:** Details of all the dynamically stable clique residues unique to the closed state (PDB_ID: 6VXX) of the SARS-CoV-2 spike protein.

| Sl. No. | Structural domains of the closed conformational state (PDB_ID:6VXX) of trimeric SARS-cov2 spike protein | Number of cliques per domain/inter domain relationship | Residue details for Cliques |
| --- | --- | --- | --- |
| 1 | NTD | 16 | A-112 A-134 A-160; B-31 B-56 B-60; B-105 B-110 B-116; B-112 B-134 B-162; A-189 A-210 A-217; C-193 C-204 C-37; C-145 C-177 C-99; C-129 C-133 C-160; A-278 A-287 A-306; B-238 B-86 B-90; B-106 B-235 B-86; C-189 C-217 C-34; C-189 C-210 C-217; C-101 C-104 C-240; B-106 B-238 B-86; A-240 A-265 A-81 |
| 2 | NTD-RBD-SD1 | 1 | A-519 A-567 C-42 |
| 3 | NTD-SD1 | 1 | B-295 B-597 B-610 |
| 4 | NTD-SD1-SD2 | 2 | B-312 B-598 B-664; C-59 C-631 C-636 |
| 5 | NTD-SD1-S2 | 1 | A-319 A-592 C-737 |
| 6 | NTD-S2 | 2 | A-739 A-745 B-319; A-737 A-740 B-319 |
| 7 | NTD-HR1 HR2 linker | 1 | B-737 B-740 C-319 |
| 8 | NTD/RBD linker-RBD | 3 | B-326 B-534 B-539; C-328 C-533 C-578; A-351 A-454 A-492 |
| 9 | NTD/RBD linker-RBD-SD1 | 3 | B-328 B-533 B-578; A-320 A-538 A-590; A-328 A-533 A-578 |
| 10 | RBD | 17 | B-376 C-405 C-408; A-376 B-409 B-416; A-370 B-473 B-489; C-342 C-368 C-374; C-472 C-480 C-488; A-457 A-461 A-465; A-502 C-437 C-506; B-370 C-421 C-456; B-370 C-456 C-473; B-375 C-403 C-505; A-369 B-421 B-456; B-437 B-508 C-505; C-347 C-436 C-509; C-474 C-480 C-488; A-342 A-374 A-434; B-347 B-436 B-509; A-342 A-374 A-436 |
| 11 | RBD-SD1 | 4 | C-535 C-554 C-583; A-544 A-564 A-579; B-330 B-544 B-579; C-544 C-564 C-579 |
| 12 | RBD-HR1 | 1 | A-984 A-988 B-383 |
| 13 | HR1/HR2 linker | 5 | C-1083 C-1088 C-1137; B-1032 B-1043 B-1048; B-1032 B-1048 B-1051; A-1028 A-1043 A-1064; A-1005 B-1005 C-1005 |
| 14 | SD1-SD2 | 4 | B-606 B-682 B-690; C-600 C-605 C-674; A-600 A-605 A-691; A-610 A-636 A-651 |
| 15 | SD1-HR1 | 1 | A-571 C-974 C-979 |
| 16 | SD1- S2 | 5 | B-855 C-589 C-592; A-568 A-574 C-854; A-740 A-857 B-592; B-775 B-864 C-665; A-592 C-737 C-855 |
| 17 | SD2 | 2 | C-662 C-671 C-697; B-641 B-654 B-691 |
| 18 | S2 | 5 | B-794 B-797 C-707; C-743 C-749 C-75; A-791 A-807 A-875; C-886 C-901 C-905; A-791 A-807 A-875 |
| 19 | S2 -HR1 | 4 | A-749 A-977 A-993; C-1000 C-741 C-966; B-762 C-1003 C-965; A-1003 A-965 C-759; A-804 A-817 A-935 |
| 20 | S2-HR1-HR1/HR2 linker | 2 | B-1011 B-731 B-955; B-1005 B-759 C-1002; |
| 21 | S2 - HR1/HR2 linker | 19 | A-1019 A-773 A-777; B-1011 B-1014 B-731; A-1005 A-759 B-1002; C-1052 C-802 C-805; B-1019 B-773 B-777; B-1109 B-718 B-915; B-1004 B-736 B-741; B-1067 B-718 B-906; A-1079 C-900 C-917; A-1107 C-886 C-904; A-918 B-1089 B-1123; C-1036 C-886 C-905 ; A-1028 A-1064 A-725; B-914 B-918 C-1089 C-1123; A-914 A-918 B-1089; A-1050 A-898 A-901; B-1052 B-802 B-927; C-1050 C-901 C-905; C-1050 C-898 C-901 |

**Table S3b:** Details of all the dynamically stable cliques unique to the partially open state (PDB_ID: 6VYB) of the SARS-CoV-2 spike protein.

| Sl. No. | Structural domains of the partially open conformational state (PDB_id:6vyb) of trimeric SARS-cov2 spike protein | Number of cliques per domain/inter domain relationship | Residue details for Cliques |
| --- | --- | --- | --- |
| 1 | NTD | 20 | B-216 B-266 B-29; A-144 A-154 A-158;B-190 B-96 B-99; B-102 B-155 B-243; B-105 B-118 B-133; B-115 B-130 B-167; B-196 B-201 B-235; A-279 A-44 A-49; A-242 A-265 A-81; C-278 C-306 C-48; C-106 C-117 C-201; C-170 C-173 C-227; A-115 A-130 A-167; B-201 B-235 B-86; B-101 B-104 B-240; A-187 A-96 A-99; C-279 C-44 C-49; C-106 C-235 C-86 ; A-299 A-308 A-313; A-101 A-240 A-265 |
| 2 | NTD-SD1 | 6 | C-318 C-612 C-621; A-318 A-612 A-621; A-562 C-224 C-38; A-562 C-225 C-38; A-295 A-597 A-608; A-299 A-315 A-597 |
| 3 | NTD-SD2 | 1 | C-294 C-635 C-685 |
| 4 | NTD-RBD | 2 | A-357 A-394 C-230; |
| 5 | NTD-S2 | 1 | A-319 C-739 C-745 |
| 6 | NTD-SD1-S2 | 1 | A-737 B-319 B-592; B-168 C-357 C-396 |
| 7 | NTD RBD linker-RBD | 1 | C-326 C-534 C-539 |
| 8 | NTD RBD linker-RBD-SD1 | 1 | B-328 B-530 B-580 |
| 9 | RBD | 16 | B-341 B-347 B-399; B-351 B-454 B-492; B-364 B-388 B-527; B-472 B-480 B-488; B-474 B-480 B-488; C-351 C-454 C-492; C-380 C-412 C-429; A-365 A-387 A-515; B-401 B-442 B-451; B-350 B-418 B-422;B-370 C-409 C-416; B-400 B-410 B-423; B-403 B-406 B-495; A-350 A-418 A-422; A-350 A-418 A-422; A-406 A-409 A-418 |
| 10 | RBD-SD1 | 1 | C-559 C-577 C-584 |
| 11 | RBD-HR1 | 1 | B-984 B-988 C-383 |
| 12 | SD1-SD2 | 3 | B-617 B-644 B-649; B-660 B-682 B-695; C-617 C-644 C-649 |
| 13 | SD1-S2 | 2 | A-788 A-873 B-699; A-737 A-740 B-592 |
| 14 | SD1-HR1 | 1 | A-567 C-976 C-979 |
| 15 | SD2 | 3 | A-662 A-671 A-697; B-663 B-673 B-682; B-673 B-682 B-695 |
| 16 | S2 | 5 | B-797 B-898 C-707; A-707 C-797 C-898; B-753 B-756 B-759; A-906 A-911 A-915; B-797 B-802 B-898 |
| 17 | S2-HR1 | 4 | B-746 B-749 B-977; B-756 B-994 C-995; B-756 C-971 C-995; C-749 C-977 C-993 |
| 18 | S2-HRI HR2 linker | 15 | B-1029 B-1053 B-877; B-897 B-900 C-1077; B-1111 B-914 C-1123; C-1029 C-1053 C-877; A-914 A-918 B-1123; A-1089 A-1123 C-914; B-1050 B-898 B-902; B-900 B-917 C-1079; B-914 B-918 C-1123; A-1052 A-802 A-805; A-1067 A-906 A-911; B-914 C-1089 C-1123; A-1052 A-805 A-878; B-1067 B-1109 B-718; A-1011 A-1014 A-731 |
| 19 | S2-HR1-HR1 HR2linker | 2 | A-1018 A-728 A-951; A-1011 A-731 A-955 |
| 20 | HR1 | 3 | B-1000 B-977 B-996; B-1002 C-1002 C-970; A-1002 B-1002 C-1002 |
| 21 | HR1- HR2 linker | 6 | B-1078 B-1102 B-1133; C-1095 C-1104 C-1115; B-1032 B-1051 B-1064; B-1032 B-1051 B-1064; C-1109 C-718 C-915; A-1031 A-1039 A-1042 |
| 22 | HR1-HR1-HR2 linker | 1 | B-1011 B-1014 B-955 |

**Table S3c:** Details of all the dynamically stable common cliques in the closed and partially open states (PDB_ID: 6VXX and 6VYB) of the SARS-CoV-2 spike protein.

| Sl. No. | Structural domains of both the closed and partially open conformational states of trimeric SARS-cov2 spike protein | Number of Cliques per domain/inter domain relationship | Residue details for Cliques |
| --- | --- | --- | --- |
| 1 | NTD | 35 | A-193 A-204 A-37; A-195 A-37 A-53; B-191 B-221 B-34; B-195 B-37 B-53; B-278 B-306 B-48; B-106 B-117 B-201; B-278 B-287 B-306; A-278 A-306 A-48; C-191 C-221 C-34; C-195 C-37 C-53; C-278 C-287 C-306; A-106 A-117 A-201; A-195 A-204 A-37; B-275 B-290 B-58; B-191 B-34 B-91; B-240 B-265 B-92; B-104 B-240 B-92 ; A-191 A-34 A-91; A-106 A-235 A-86 |
| 2 | NTD-RBD | 3 | B-322 B-540 B-549; C-322 C-540 C-549; A-322 A-540 A-549 |
| 3 | NTD-RBD-SD1 | 5 | A-559 A-563 C-43; A-42 B-519 B-567; B-43 C-559 C-563; B-42 C-519 C-567; A-43 B-559 B-563; |
| 4 | NTD-SD1 | 2 | B-318 B-612 B-621; B-299 B-315 B-597 |
| 5 | NTD/RBD linker-RBD | 1 | A-326 A-534 A-539 |
| 6 | RBD | 33 | B-342 B-374 B-434; B-454 B-457 B-467; B-457 B-461 B-465; C-364 C-388 C-527; C-454 C-457 C-467; C-457 C-461 C-465; A-454 A-457 A-467; B-342 B-374 B-436; B-353 B-398 B-423; B-392 B-395 B-515; B-438 B-442 B-509; B-438 B-442 B-507 ; B-439 B-443 B-507; C-342 C-374 C-434; C-350 C-418 C-422; C-353 C-398 C-423; C-392 C-395 C-515; C-400 C-410 C-423; C-403 C-406 C-495; C-438 C-442 C-509; C-438 C-442 C-507; C-439 C-443 C-507; A-347 A-436 A-509; A-353 A-398 A-423; A-392 A-395 A-515; A-403 A-406 A-495; A-438 A-442 A-509; A-438 A-442 A-507; A-439 A-443 A-507; B-353 B-400 B-423; C-342 C-374 C-436; C-353 C-400 C-423; A-353 A-400 A-423 |
| 7 | RBD-SD1 | 4 | C-330 C-544 C-579; A-559 A-577 A-584; B-559 B-577 B-584; B-544 B-564 B-579 |
| 8 | SD1-SD2 | 1 | B-600 B-605 B-674 |
| 9 | SD2 | 1 | B-662 B-671 B-697 |
| 10 | s2 | 5 | C-797 C-802 C-898; B-797 B-802 B-882; C-797 C-802 C-882; A-797 A-802 A-882; B-903 B-913 B-917 |
| 11 | S2- HR1 | 2 | B-804 B-817 B-935; C-804 C-817 C-935 |
| 12 | S2-HR1/HR2 linker | 10 | A-1050 A-901 A-905; A-886 A-904 B-1107; A-900 A-904 B-1094; C-1067 C-1109 C-718; B-1052 B-802 B-805; B-1054 B-816 B-819; C-1054 C-816 C-819; A-1029 A-1053 A-877; A-1054 A-816 A-819; A-1077 C-897 C-900 |
| 13 | S2 - HR1- HR1-HR2 linker | 1 | C-1011 C-1014 C-731 C-955 |
| 14 | HR1-HR2 linker | 14 | B-1029 B-1033 B-1053; B-1029 B-1033 B-1053; B-1095 B-1104 B-1115; C-1029 C-1033 C-1053; C-1032 C-1043 C-1048; C-1078 C-1102 C-1133; C-1082 C-1126 C-1132; A-1029 A-1033 A-1053; A-1032 A-1043 A-1048; A-1032 A-1048 A-1051;A-1067 A-1109 A-718; C-1032 C-1048 C-1051; A-1082 A-1126 A-1132; A-1095 A-1104 A-1115 |

**Table S4**: Details of all the dynamically stable unique interfacial clique residues in the

closed (PDB_ID: 6VXX) and partially open state conformations (PDB_ID: 6VYB) of the SARS-CoV-2 spike protein.

|  | Domains | Closed Structure of SARS-CoV-2  (PDB_ID: 6VXX) | | Partially open structure of SARS-CoV-2 (PDB_ID: 6VYB) | |
| --- | --- | --- | --- | --- | --- |
|  |  | No. of interfacial cliques | Details of Interfacial Cliques | No. of interfacial cliques | Details of Interfacial Cliques |
| 1 | NTD-SD1 | 0 | 0 | 3 | A-224 A-38 B-562; A-562 C-224 C-38; A-562 C-225 C-38 |
| 2 | NTD-S2 | 3 | B-737 B-740 C-319; A-739 A-745 B-319; A-737 A-740 B-319; | 1 | A-319 C-739 C-745 |
| 3 | NTD-RBD | 0 | 0 | 2 | A-357 A-394 C-230; B-168 C-357 C-396 |
| 4 | NTD-SD1-S2 | 0 | 0 | 1 | A-737 B-319 B-592 |
| 5 | RBD | 9 | B-376 C-405 C-408; A-376 B-409 B-416; A-370 B-473 B-489; A-502 C-437 C-506; B-370 C-421 C-456; B-370 C-456 C-473; B-375 C-403 C-505; A-369 B-421 B-456; B-437 B-508 C-505 | 1 | B-370 C-409 C-416 |
| 6 | RBD-HR1 | 1 | A-984 A-988 B-383 | 1 | B-984 B-988 C-383 |
| 7 | NTD-RBD-SD1 | 1 | A-519 A-567 C-42 | 0 | 0 |
| 8 | NTD-SD1-S2 | 1 | A-319 A-592 C-737 | 0 | 0 |
| 9 | SD1-S2 | 4 | B-855 C-589 C-592; A-568 A-574 C-854; A-740 A-857 B-592; A-592 C-737 C-855 | 2 | A-788 A-873 B-699; A-737 A-740 B-592 |
| 10 | SD1-HR1 | 1 | A-571 C-974 C-979 | 1 | A-567 C-976 C-979 |
| 11 | SD2-S2 | 1 | B-775 B-864 C-665 | 0 | 0 |
| 12 | S2 | 1 | B-794 B-797 C-707 | 2 | A-707 C-797 C-898; B-797 B-898 C-707 |
| 13 | S2-HR1 | 2 | A-1003 A-965 C-759; B-762 C-1003 C-965; | 2 | B-756 B-994 C-995; B-756 C-971 C-995 |
| 14 | S2- HR1/HR2 linker | 4 | A-1079 C-900 C-917; A-914 A-918 B-1089; B-914 B-918 C-1089 C-1123; A-918 B-1089 B-112 | 7 | B-897 B-900 C-1077; B-1111 B-914 C-1123; A-914 A-918 B-1123; A-1089 A-1123 C-914; B-900 B-917 C-1079; B-914 B-918 C-1123; B-914 C-1089 C-1123 |
| 15 | S2-HR1-HR1/HR2 linker | 3 | A-1005 A-759 B-1002; B-1005 B-759 C-1002; A-1107 C-886 C-904; | 0 | 0 |
| 16 | HR1 | 0 | 0 | 2 | A-1002 B-1002 C-1002; B-1002 C-1002 C-970 |
| 17 | HR1/HR2 linker | 1 | A-1005 B-1005 C-1005 | 0 | 0 |
